# Boosting species evenness, productivity and weed control in a mixed meadow by promoting arbuscular mycorrhizas

**DOI:** 10.3389/fpls.2024.1303750

**Published:** 2024-02-08

**Authors:** Ludovica Oddi, Veronica Volpe, Gennaro Carotenuto, Mara Politi, Elena Barni, Andrea Crosino, Consolata Siniscalco, Andrea Genre

**Affiliations:** Department of Life Sciences and Systems Biology, University of Turin, Turin, Italy

**Keywords:** arbuscular mycorrhizas, forage plants, fungal inoculum, grass-legume seed mixture, pastoral value, mixed meadow, chito-oligosaccharides, sustainable agriculture

## Abstract

Lowland meadows represent aboveground and belowground biodiversity reservoirs in intensive agricultural areas, improving water retention and filtration, ensuring forage production, contrasting erosion and contributing to soil fertility and carbon sequestration. Besides such major ecosystem services, the presence of functionally different plant species improves forage quality, nutritional value and productivity, also limiting the establishment of weeds and alien species. Here, we tested the effectiveness of a commercial seed mixture in restoring a lowland mixed meadow in the presence or absence of inoculation with arbuscular mycorrhizal (AM) fungi and biostimulation of symbiosis development with the addition of short chain chito-oligosaccharides (CO). Plant community composition, phenology and productivity were regularly monitored alongside AM colonization in control, inoculated and CO-treated inoculated plots. Our analyses revealed that the CO treatment accelerated symbiosis development significantly increasing root colonization by AM fungi. Moreover, the combination of AM fungal inoculation and CO treatment improved plant species evenness and productivity with more balanced composition in forage species. Altogether, our study presented a successful and scalable strategy for the reintroduction of mixed meadows as valuable sources of forage biomass; demonstrated the positive impact of CO treatment on AM development in an agronomic context, extending previous observations developed under controlled laboratory conditions and leading the way to the application in sustainable agricultural practices.

## Introduction

In an agricultural context, management intensification is a major driver of biodiversity loss (Sala et al., 2000; Foley et al., 2005; Culman et al., 2010). Despite their importance, lowland meadows are critically endangered habitats in many European countries, because of hydrological perturbation and land-use change leading to the decline of meadow cover area and distribution, and the alteration of their species composition (Poschlod et al., 2005). The category of ‘agricultural grasslands’ includes silage and hay fields, pastures under intensive production, and semi-natural grasslands. Over the last decades, silage made from arable crops such as maize has become much more widely adopted in irrigated areas than hay production, due to economic reasons, with serious consequences on biodiversity at different trophic levels, such as high mortality of ground-nesting birds and resource removal for other taxa, especially pollinators (Stoate et al., 2009).

Furthermore, the intensive use of pesticides, herbicides, and fertilizers had a strong impact on soil biodiversity, causing cultivated varieties to become partially reluctant to the development of beneficial interactions with soil-borne microorganisms (Duhamel and Vandenkoornhuyse, 2013). Moreover, humans significantly altered the composition of many natural plant communities through the deliberate or accidental introduction of exotic species (Van der Putten et al., 2007; Milardi et al., 2020), reducing biodiversity and compromising ecosystem functionality (Griffiths and Philippot, 2013; Agathokleous et al., 2020). Several studies reported that repeated monoculture cropping, the inclusion of non-mycorrhizal crops within rotation and fallowing affect mycorrhizal activity and decrease crop yields (Baltruschat and Dehne, 1988; Hetrick et al., 1996; Douds et al., 1997; Arihawa and Karasawa, 2000; Harrier and Watson, 2004).

In addition to their importance for biodiversity, lowland meadows also provide significant ecosystem services: water retention and filtration, forage production, soil protection from erosion and contribution to its fertility, and potential for carbon sequestration (Hopkins and Holz, 2006; Stoate et al., 2009). Finally, lowland grasslands also have a significant aesthetic value and support recreation in the countryside (Boval and Dixon, 2012).

Besides its ecological implications, the biodiversity of lowland meadows also provides several benefits for farmers. Indeed, the presence of functionally different plant species improves forage quality and nutritional value. In this frame, French (2017) demonstrated that forage from species-rich grasslands contained more protein, phosphorus, potassium, and calcium than cereals and conventional hay. Moreover, higher biodiversity, especially in terms of functional dispersion and species evenness, enhances the productivity of plant communities and consequently reduces available space for the establishment of weeds and alien species, representing an effective and low-cost strategy for weed control (Sanderson et al., 2012; Suter et al., 2017).

For all these reasons, the reintroduction of lowland meadows has become a major goal in the last two decades, in particular in cattle farms where both animal health and dairy product quality take advantage of hay-based feeding. Moreover, grasslands management requires lower energy and fertilizer input than cereals, thus supporting more efficient, sustainable, and adaptable agricultural practices with a reduced environmental impact, especially in those farms where grasslands were replaced decades ago by cereal monoculture. Lastly, permanent grasslands constitute no tillage areas, with a major positive impact on the maintenance of the microbial communities in the soil (Guldberg et al., 2022).

When comparing the results of different agronomic techniques in restoring meadow biodiversity, Sengl et al. (2017) found that sod transplantation and hay transfer were more successful than seeding. However, sod transplantation requires the destruction of valuable grassland habitat, whereas hay transfer is often associated with low seed germination rate; furthermore, both approaches require the availability of source sites and are relatively expensive. As a consequence, the use of commercial seed mixtures remains the most convenient technique in highly productive areas, albeit its weak effectiveness in biodiversity restoration needs to be improved.

This goal can be achieved by exploiting the potential of soil microbial biodiversity in influencing plant diversity in grasslands. Although the functional implications for restoration require further investigations, the soil communities of diverse grasslands tend to be dominated by decomposer fungal communities and mycorrhizal networks (Bardgett, 1996; Smith et al., 2003; Walker et al., 2004). In particular, the latter are believed to play a key role in driving ecosystem processes, such as nutrient cycling and plant productivity, as well as controlling community composition and structure during the early phases of succession (Walker et al., 2004).

Arbuscular mycorrhiza (AM), involving root colonization by specialized soil fungi belonging to Glomeromycotina, represents the most widespread plant symbiosis (Genre et al., 2020). Indeed, around 72% of plant species - including the vast majority of forage and crop plants - host AM fungi in their root tissues. By exploring a larger volume of soil, the extraradical mycelium grants roots a more efficient access to mineral nutrients (namely phosphorus and nitrogen) and water (Smith et al., 2010). Furthermore, AM symbiosis increases plant tolerance to water stress and reinforces defense against pathogens (Shi et al., 2023). In return, AM fungi are fed with a percentage of plant metabolites, such as sugars and lipids, that are essential for the completion of their life cycle, in a truly mutualistic relationship (Smith and Read, 2008; Jiang et al., 2017; Keymer et al., 2017; Luginbuehl et al., 2017).

In this frame, due to their broad host range, different AM fungi have been shown to provide different degrees of benefits to different host species, overall generating a complex and versatile exchange mechanism that influences the structure of plant communities (van der Heijden et al., 2015) and has been likened to a biological market (Wyatt et al., 2014). Generally, AM fungal diversity was observed to be positively related to plant species diversity and productivity, due to a relaxation of plant competitive interactions and to the promotion of subordinate species (Bardgett, 1996; Walker et al., 2004). Moreover, mycorrhizas may promote seedling establishment with important advantages in the early stages of development of the plant community (van der Heijden, 2004).

Concerning forage species, evidence is accumulating that their symbiotic status is a basic requirement for sustainable feed production and any advance in the optimization of this symbiotic system may lead to an improvement in forage productivity and nutritional properties (Baslam et al., 2014; Hack et al., 2019). Therefore, due to their ecological and nutritional functions, AM fungi must be seen as an important biotechnology in sustainable agriculture, leading to improved agricultural management of soil and crops (Lanfranco et al., 2016) and eventually increasing the efficiency of lowland meadow restoration.

Currently, the most commonly introduced change in soil management to promote AM associations has been to limit acknowledged harmful practices, such as deep tillage and fungicide use, and introduce AM fungal inocula in crop fields. The main drawback in this approach is related to the lack of information on how a commercial AM fungal strain is performing with each crop species and variety; how it adapts to local soil and climate conditions; how it competes with the native AM fungal community (Douds et al., 2005; Gosling et al., 2006). Furthermore, due to the prolonged selection of crop cultivars for their productivity in highly fertilized soils (with rather limited attention to their symbiotic associations), many cultivated species are recalcitrant to engage in AM symbiosis and AM inoculation alone may have limited effects on their mycorrhizal status (Duhamel and Vandenkoornhuyse, 2013).

An innovative approach to address this problem has been to boost pre-symbiotic signaling by exogenously treating the plants with fungal molecules that promote symbiosis development. Such mycorrhizal factors (or myc-factors) are known to be released by symbiotic fungi and trigger cellular and molecular responses that accelerate symbiosis establishment (Volpe et al., 2023). In this frame, short chain chito-oligosaccharides (COs) have been shown to be recognized as myc-factors in a wide range of host plants, including legumes and monocots (Genre et al., 2013; Sun et al., 2015) and their use as promoters of AM establishment paved the way to possible applications in sustainable agriculture and pasture management (Volpe et al., 2020; Volpe et al., 2023).

In this research we tested the effectiveness of a commercial seed mixture in restoring a lowland mixed meadow in an area of the Po plain, where grasslands have been replaced by cereals for two decades. Furthermore, we evaluated the impact of a commercial AM inoculum combined with myc-factor treatment on the composition of the plant community and its productivity.

## Materials and methods

### Study area location and soil properties

The present study was carried out in South-Western Piedmont (Italy), in a 5 ha experimental field located in Monasterolo di Savigliano (Lat 44.6894147, Long 7.6196066) (**Supplementary Figure S1**). In order to prevent soil heterogeneity caused by the edge effect and uneven water distribution during flow irrigation, we decided to locate our experimental area in the central part of the field (**Supplementary Figure S1**). Soil homogeneity in the selected area was confirmed by the analysis of soil chemical properties (by Camera di Commercio di Torino; www.lab-to.camcom.it) in 5 sampling points distributed along the two diagonals crossing the experimental area.

The same sampling points were used to evaluate the presence and activity of the native AM fungal community at time zero. To this aim, 20x20x20 cm soil samples were collected from the soil surface. Soil samples were pooled and mixed with 20% sterilized sand (0.4-0.8 mm; Valle Po, Revello, CN, Italy) and used to fill 5 plastic pots (biological replicates), where a 2 cm layer of perlite had previously been introduced for draining. Two *Medicago sativa* seeds were planted in each pot and plants were grown in a greenhouse, supplied with tap water as needed and fertilized once per week with a modified Long Ashton nutrient solution (Hewitt, 1966) with a low phosphate content (3.2 µM), to allow AM development. After 4 months, the roots were sampled from each biological replicate and used to quantify AM colonization according to Trouvelot et al. (1986), as described below.

### Experimental setup and sampling design

The experimental area (**Supplementary Figure S2**) was split into three plots, corresponding to an untreated control (CTR, 12.400 m^2^), a mycorrhizal (MYC, 23.800 m^2^), where seeds were coated with a commercial inoculum, and a CO-treated mycorrhizal plot (MYC+CO, 12.800 m^2^), where seed coating was added with a mix of short chain COs (see below).

All samples were collected using the sampling scheme presented in **Supplementary Figure S2**. For each treatment six circular sampling areas (radius = 4 m) were identified and distributed at regular distances (15 m) along a longitudinal line in the central part of the three experimental plots (**Supplementary Figure S2**). Within each circular sampling area, soil and plant samples were randomly collected, avoiding previous sampling points. Sample collection was carried out immediately before mowing in three time points covering two productive seasons: April 2017/2018, July 2017/2018 and October 2017/September 2018. Two additional samplings devoted to AM colonization were carried out during winter (December 2016 and February 2018).

### Seed mixture and sowing

The field was plowed and sown in October 2016,with a mixture of commercial native species (**Supplementary Figure S3**), composed of *Dactylis glomerata* cv. Dactyna (40%; 12.3 kg/ha), *Festuca arundinacea* cv. Kora (20%; 13.8 kg/ha), *Trifolium pratense* cv. Krinya o Nike (10%; 3.7 kg/ha), *Poa pratensis* cv. Balin (5%; 0.6 kg/ha), *Medicago sativa* cv. Vogherese Padus (20%; 10.9 kg/ha) and *Festulolium* cv. Becva (5%; 3.7 kg/ha).

### AM inoculum and CO treatment

The commercial microbial inoculum Micosat F (**Supplementary Table S1**; CCS Aosta S.r.l.) was used in MYC and MYC+CO plots. In more detail, the seed mixture used for the MYC plot was coated with 1 kg/ha of Micosat F by mixing the inoculum powder with the seed mixture inside the hopper tank, immediately before sowing. For CO treatments, 100g/ha of powder containing a mixture of short chain chito-oligosaccharides (CO2-CO5; Zhengzhou Sigma Chemical Co., Ltd. Zhengzhou, Henan, China) were added to the seed coating, alongside the inoculum. A second CO treatment was applied in November 2017, by spraying a 1 g/L CO solution in water on the meadow, using a tractor-operated bar sprayer. Specifically, we sprayed 100 L/ha of CO solution for the MYC+CO plot and 100 L/ha of water for MYC and CTR plots.

### Biomass and plant community analyses

To estimate forage productivity, aboveground plant biomass was collected from each sampling point by mowing all the plant material at the soil surface inside a 30x30 cm wooden frame randomly located within the circular sampling area, avoiding previous sampling points. After collection, biomass was immediately transferred to the lab and manually sorted into grasses, forbs and weeds. All the sorted samples were then oven-dried at 65°C for 24 hours and weighed to measure the dry mass.

Plant community composition was analyzed through vegetation surveys based on vertical point quadrat transects (Daget and Poissonet, 1971). Three 15-meter long transects were located alternately between sampling points and records of the plant species contacts were performed every 0.50 m for a total of 30 contact points per transect and 90 contact points per treatment.

These data were used to calculate alpha diversity considering the total number of plant species and evenness Pielou index.

The pastoral value (PV) of the meadows under different experimental conditions (see below) was calculated according to the equation (Daget and Poissonet, 1971):


$$PV=\frac{\sum_{i=1}^{n}SC_{i}\times ISQ_{i}}{5}$$

where SC_i_ is the specific contribution (i.e. the percentage of each species in the total vegetation as derived from the Daget-Poissonet method), and ISQ_i_ is the Index of Specific Quality (ranging between 0 and 5 as shown in **Supplementary Table S2**), depending on the preference, morphology, structure, and productivity of the plant species (Cavallero et al., 1992; Cavallero et al., 2002; Cavallero et al., 2007). The resulting PV ranges between 0 and 100.

### Quantitative analysis of AM colonization

Roots were isolated by rinsing each soil sample with tap water. Six biological replicates consisting of at least 1 m of root were analyzed for each sampling point and used for morphological and quantitative analyses. Root samples were carefully cleared of adhering soil debris and then stained in 0.1% (W/V) cotton blue in lactic acid for at least 12 h, destained in freshly prepared lactic acid solution for 3-4 times and cut into 1 cm-long segments. The segments were then placed on microscope slides and mounted in glycerol for observation in bright-field microscopy. AM colonization level was quantified in each segment according to Trouvelot et al. (1986).

### Statistical analyses

Statistical analysis and figure creation were performed using R 4.3.0 (R Core Team, 2023) and the packages ‘betapart’, ‘devtools’, ‘dplyr’, ‘ggplot2’, ‘ggtern’, ‘indicspecies’, ‘mass’, ‘multcomp’, ‘pairwiseAdonis’, ‘Rmisc’, and ‘vegan’. In the case of data showing normal distribution (i.e., aboveground plant biomass), the differences among treatments were tested for significance using generalized linear models (GLMs) followed by Tukey’s HSD *post hoc* test for multiple comparisons, with a single-step method to calculate adjusted p-values (95% CI). When data were not normally distributed (i.e., frequency of AM colonization, species richness, Pielou index, biomass composition, and pastoral value) the non-parametric Kruskal-Wallis test by rank was performed, followed by the pairwise Wilcoxon rank sum tests and p-value Bonferroni adjustment method, to calculate pairwise comparisons between group levels with corrections for multiple testing.

To test the differences in plant community structure and composition multivariate analyses were performed. Firstly, we calculated the beta dispersion of Bray-Curtis dissimilarity matrices and tested it for homogeneity among treatments. Afterwards, since the assumption of homogeneity was verified, we performed a permutational multivariate analysis of variance (PERMANOVA) using the wrapper function ‘pairwise.adonis’ for multilevel pairwise comparison and p-value Bonferroni adjustment method.

## Results

### Soil properties

A preliminary chemical analysis of 5 samples was shown in the **Supplementary Figure S1**, with an average of a pH of 6.3, a 7.36 C/N ratio, 20.4 and 35.2 g/kg content in organic carbon and organic matter, respectively. The soil resulted to be particularly rich in assimilable nitrogen (0.3%) and phosphate (64.8 mg/kg), a condition that does not favor AM development (Balzergue et al., 2013; Wang et al., 2017). Nevertheless, our microcosm experiment resulted in a high level of root colonization in the trap plants of *M. sativa*, with a mycorrhizal frequency (F%) of 50% and arbuscular abundance (A%) close to 10% (**Supplementary Figure S4**).

### AM colonization

**Figure 1:**
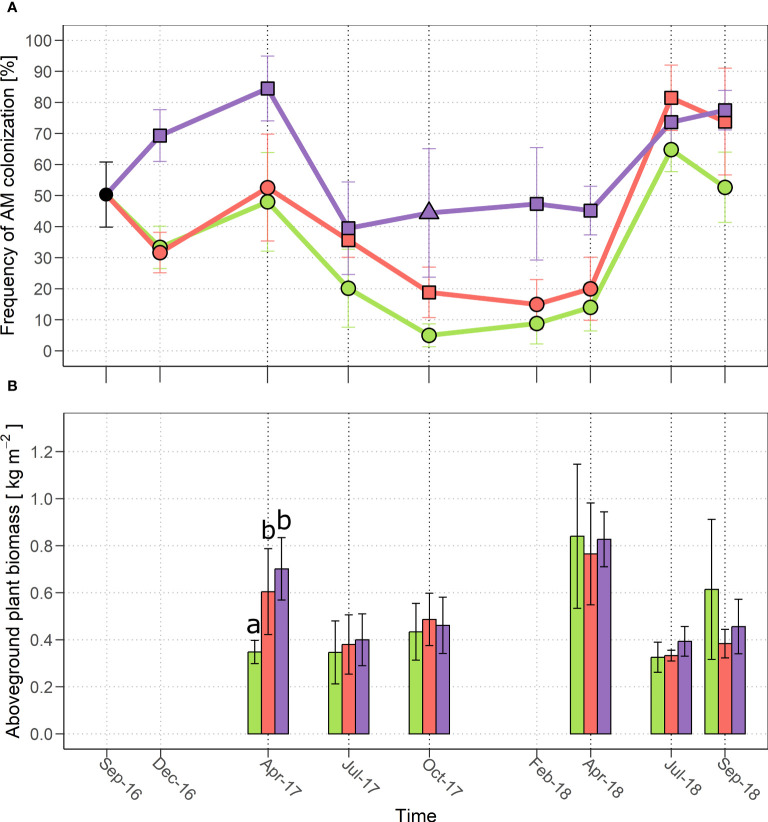
**(A)** Frequency of AM colonization and **(B)** aboveground plant biomass productivity from (October 2016) to September 2018 in the three treatments: control (green), MYC (orange) and MYC+CO (purple). In **(A)** different symbols indicate statistically significant differences among treatments (*p*< 0.05) within a single time point; in **(B)** significant differences among treatments are indicated by different letters. Error bars represent ± SD.

The quantification of symbiotic fungal structures in root samples from each experimental plot over time is presented in **Figure 1A**. The most apparent feature, present in all the three treatments, is a seasonal cycle of mycorrhization intensity, with a progressive increase through winter, a peak in spring-early summer and a subsequent drop. The evident shift in the colonization peak from April (2017) to July (2018) should be ascribed to climate variability between years. In particular, the spring of 2017 was rather dry and warm, whereas relatively low temperatures were registered until May 2018, with abundant rain (**Supplementary Figure S5**).

MYC treatment did not significantly change this seasonal pattern compared to CTR plants, even if a limited (and statistically significant) increase in root colonization was recorded over the summer of both 2017 and 2018, suggesting that microbial inoculation had a positive effect on the AM community and symbiosis development.

By contrast, MYC+CO treatment produced a major increase in AM colonization compared to both MYC and CTR conditions, throughout the initial phase of the project, from December 2016 to April 2017; the samples from July 2017 are the only exception, as the general summer decline lowered root colonization to a level that was equivalent to the other two experimental conditions. In more detail, the most important increase was recorded immediately after sowing, in December 2016 and April 2017, when root colonization in MYC+CO plants exceeded 80%, compared to values ​​close to 50% for CTR and MYC plants. The MYC+CO treatment granted a higher level of mycorrhization also between October 2017 and April 2018 (constantly above 40%, compared to values between 10% and 20% for MYC and CTR). Due to the progressive increase in AM development from 2017 to 2018, all experimental plots reached a comparable level of colonization in July 2018 and a significantly higher colonization level (above 70%) was maintained in MYC and MYC+CO compared to CTR plants (50%) in September 2018.

Lastly, besides the recursive seasonal cycle and irrespectively of the plot treatment, a general increase in AM colonization was observed between the first season (July-October 2017) and the second one (July-October 2018) across all samples. The occurrence of this increase also in the CTR plot, suggests that the mixed meadow management had a positive impact, over time, on the activity of the native AM community also in the absence of any additional treatment.

### Plant productivity

Concerning plant productivity, in 2017 MYC (2.98 ± 0.57 kg m^-2^) and MYC+CO (2.80 ± 0.21 kg m^-2^) produced a significantly higher (*p*< 0.01) amount of aboveground biomass compared to CTR (2.30 ± 0.19 kg m^-2^). Furthermore, a significantly higher production (*p*< 0.001) was observed in MYC (+0.25 kg m^-2^) and MYC+CO (+0.35 kg m^-2^) compared to CTR plants in April 2017 (**Figure 1B**). Nevertheless, this pattern was not recorded in 2018, when the aboveground plant productivity did not differ significantly among treatments.

### Plant community composition

**Figure 2:**
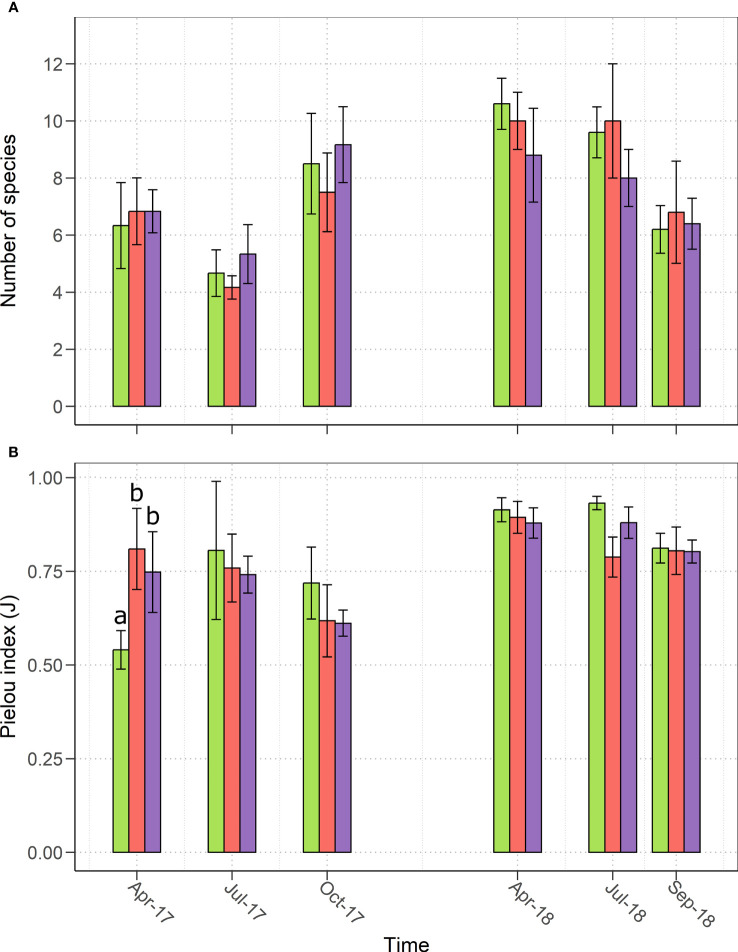
**(A)** Species richness and **(B)** Pielou evenness index **(J)** observed from April 2017 to September 2018 in the three treatments: control (green), MYC (orange) and MYC+CO (purple); significant differences among treatments are indicated by different letters. Error bars represent ± SD.

The analysis of community alpha diversity showed that the total number of plant species did not significantly differ among the three treatments in 2017 and 2018 (**Figure 2A**). Concerning the distribution of abundances across the species in the community, a significant (*p<* 0.05) difference was observed after sowing in April 2017, when MYC and MYC+CO showed greater values of Pielou index (**Figure 2B**) suggesting the absence of a strongly dominating species, which instead was present in the control meadow, where *Festulolium* constituted more than half of the meadow in terms of relative percentage cover. In general, we observed an increase in the number of species as well as in the community evenness from 2017 to 2018, and a homogenization of the samples showed by a decrease in the standard deviation values (**Figure 2A, B**).

The overall plant community composition significantly differed among all treatments in both 2017 (R^2^ = 0.47; F = 6.66, *p* = 0.001) and 2018 (R^2^ = 0.62; F = 9.72, *p* = 0.001). In particular, the MYC+CO community was significantly different from both CTR (2017: *p* = 0.01; 2018: *p* = 0.04) and MYC (2017: *p* = 0.02; 2018: *p* = 0.03) treatments throughout the two years of the study.

**Figure 3:**
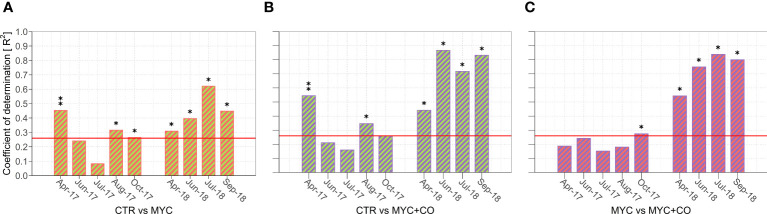
**(A-C)** Results of the pairwise comparison showing the proportion of variance (R^2^) in the plant community composition (response variable) explained by the treatment factor (explanatory variable). **(A)** shows the differences between the plant community composition of CTR and MYC, **(B)** between CTR and MYC+CO, and **(C)** between MYC and MYC+CO. The bars overcoming the red horizontal line correspond to significant differences between plant communities: (*) p-value< 0.05 and (**) p-value< 0.001.

As summarized in **Figure 3A-C**, the seasonal pattern in 2017 revealed significant differences in plant species composition among treatments at the beginning (April) and at the end (September-October) of the growing season, whereas plant community composition did not differ significantly among treatments during summer (June-July). By contrast, 2018 was characterized by a constant statistically significant difference among treatments. Moreover, pairwise comparison highlighted that, in 2017, these differences were mainly due to significant differences between CTR and the two inoculated treatments (MYC and MYC+CO) (**Figure 3A, B**), while in 2018 the three treatments showed consistent differences that were always –significant (**Figures 3A-C**).

The indicator species analysis (**Table 1**) showed that, in 2017, *Festulolium* was significantly associated with CTR, whereas *Medicago sativa*, *Trifolium pratense* and were significantly associated with MYC+CO treatment. Remarkably, indicator species association changed in 2018, with *Festulolium* significantly associated with MYC, and *Dactylis glomerata* and *Poa trivialis* associated with MYC+CO.

**Table 1:** Results of the indicator species analyses showing the species that were significantly associated with each treatment in 2017 and 2018; only significant *p-values* were reported.

Plant species	CTR		MYC		MYC+CO	
	2017	2018	2017	2018	2017	2018
*Festulolium*	0.0004			0.0144		
*Medicago sativa*					0.0074	
*Trifolium pratense*					0.0069	
*Dactylis glomerata*						0.0007
*Poa trivialis*						0.0085

### Biomass composition and pastoral value

**Figure 4:**
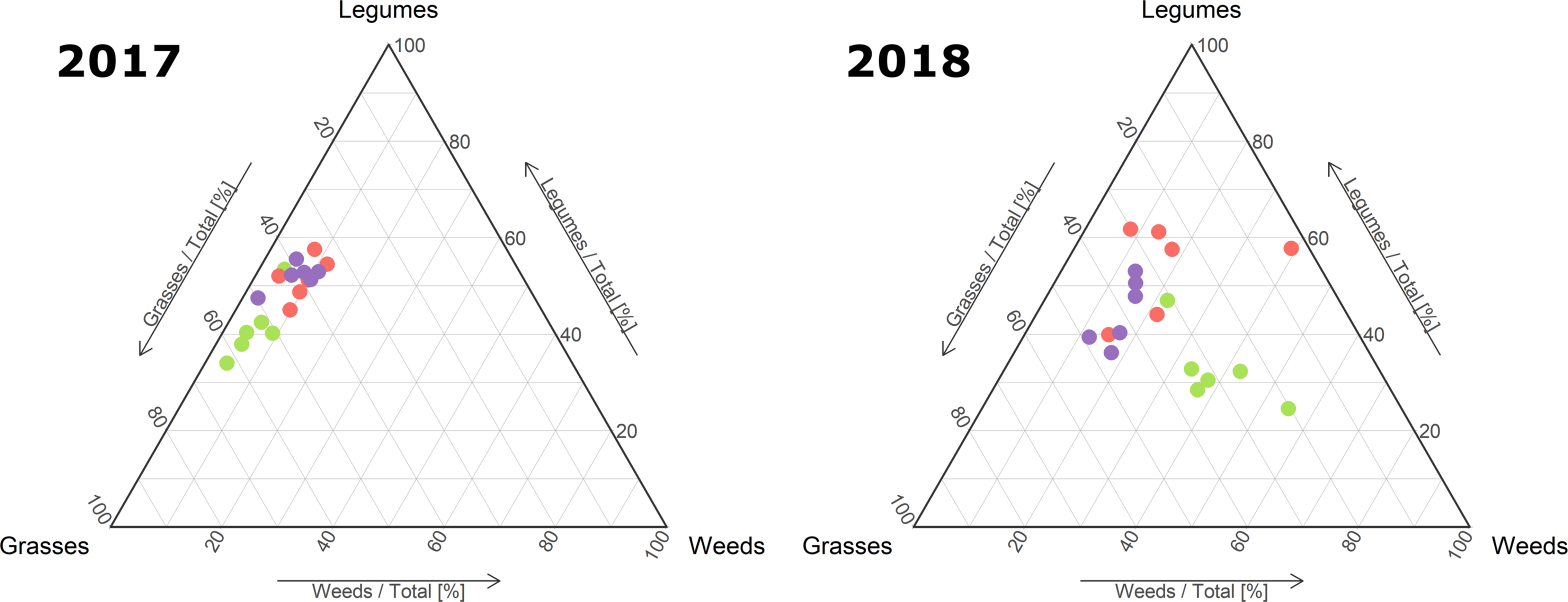
Ternary plot of grass, legume and weed contribution [%] to the forage biomass during 2017 and 2018 in the three treatments: CTR (green), MYC (orange) and MYC+CO (purple).

The analysis of the biomass composition in terms of plant functional groups highlighted that grass, legume and weed contribution to the aboveground biomass changed in the two seasons (**Figure 4**). In more detail, grass (χ^2^ = 9.58, *p*< 0.01) and legume (χ^2^ = 6.47, *p*< 0.05) contribution to the plant biomass composition in 2017 showed significant differences between treatments. Moreover, in 2018 significant differences among treatments were recorded for grass (χ^2^ = 7.38, *p*< 0.05), legume (χ^2^ = 9.98, *p*< 0.01) and weed (χ^2^ = 9.55, *p*< 0.01) contribution to the biomass. Overall, in 2017 the plant biomass produced in the MYC+CO treatment showed a significantly lower (*p*< 0.05) percentage of grasses compared to CTR and MYC, whereas the percentage of legume biomass was significantly higher (*p*< 0.05) in both MYC and MYC+CO compared to CTR (**Table 2**). In 2018, CTR contained the highest percentage of weeds showing significant differences (*p<* 0.01) with MYC+CO. Lastly, CTR showed the lowest legume contribution to the plant biomass, with significant differences (*p*< 0.05) with both MYC and MYC+CO (**Table 2**). In conclusion, the MYC+CO treatment displayed a better balance between grass and legume percentage contribution to plant biomass productivity, and the lowest percentage of weeds.

**Table 2:** Mean annual values ( ± standard deviation) of the percentage contribution to the forage biomass of three plant functional groups during 2017 and 2018; for each functional group, values in bold are significantly different from the other treatments in the same year.

Year	Treatment	Grasses [%]	Legumes [%]	Weeds [%]
2017	CTR	**53.22 ± 6.86**	**41.38 ± 6.58**	5.40 ± 1.91
	MYC	39.61 ± 4.74	51.51 ± 4.38	8.87 ± 2.56
	MYC+CO	40.51 ± 4.80	52.07 ± 2.64	7.42 ± 3.07
2018	CTR	29.43 ± 5.56	**32.60 ± 7.65**	37.97 ± 10.82
	MYC	27.10 ± 13.84	53.73 ± 9.34	19.18 ± 10.71
	MYC+CO	**40.41 ± 6.41**	44.54 ± 6.86	**15.04 ± 2.16**

**Figure 5:**
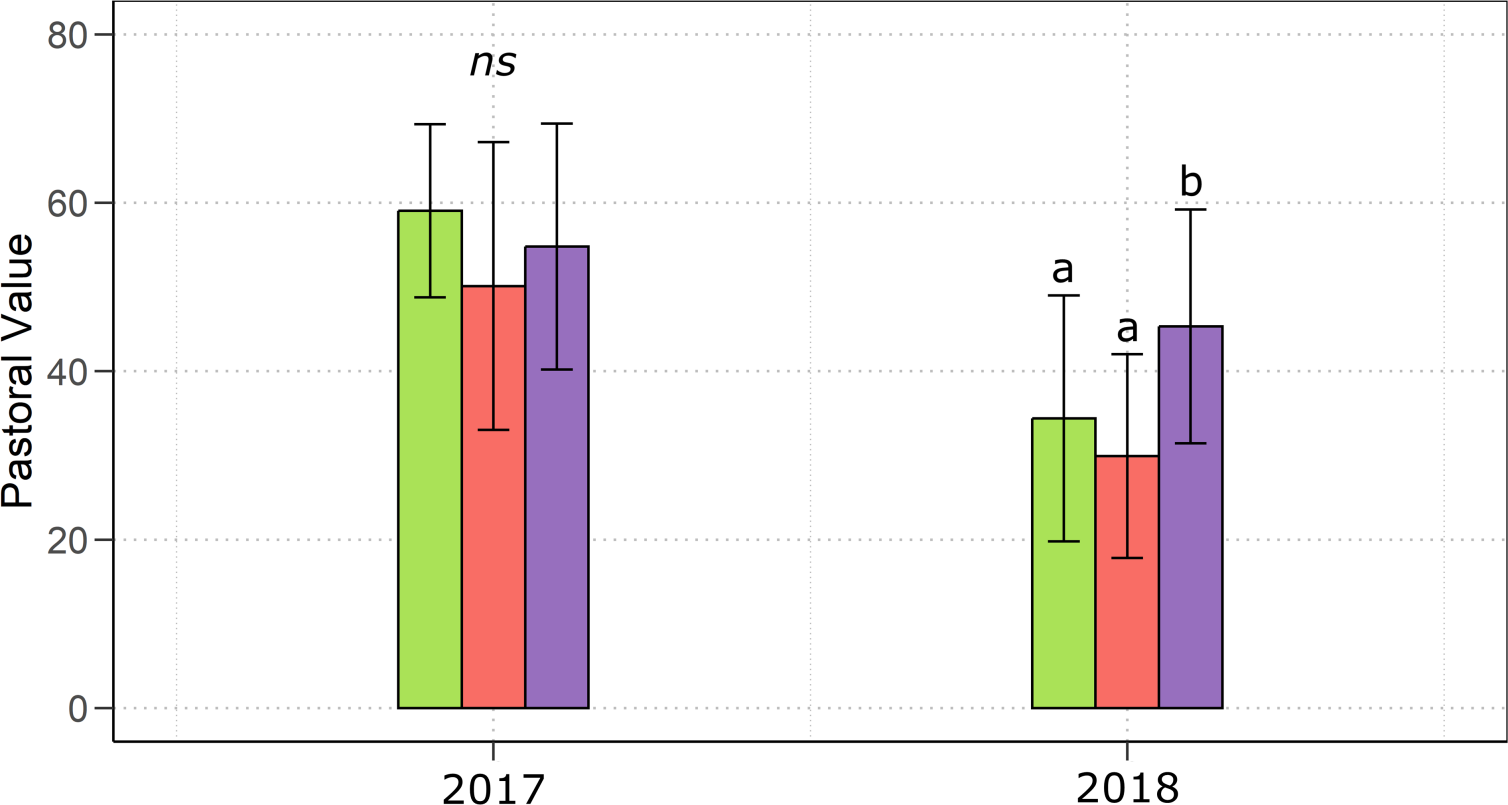
Annual mean pastoral value of the mixed meadows during the growing season 2017 and 2018 under the three different treatments: control (green), MYC (orange) and MYC+CO (purple). Error bars represent ± SD. Different letters indicate statistically significant differences; ns = non significant.

The above-mentioned differences in the plant community composition in terms of species evenness and functional group contribution to the aboveground biomass, led to significant differences in the pastoral value of the forage collected during the growing season (**Figure 5**), which, in 2018, was significantly (*p*< 0.01) higher in MYC+CO compared to CTR and MYC.

## Discussion

The current study provides first evidence that the combined application of CO and a mycorrhizal inoculum during sowing of a mixed meadow promotes AM symbiosis under field conditions, at least in the first year of application, in line with previous studies in controlled laboratory conditions (Volpe et al., 2020; Volpe et al., 2023), and has a major impacts on plant community. However, mixed meadow management had a positive impact, over time, on the activity of the native AM community also in the absence of any additional treatment. Previous studies have demonstrated the CO-dependent promotion of AM under controlled conditions (Volpe et al., 2020). More recent investigations studies, carried out in the model legume *Medicago truncatula*, revealed that the observed acceleration of AM development in CO-treated plants was correlated with the stimulation of pre-symbiotic responses in the host root, including the regulation of gene expression and the triggering of cellular responses that are known to take place during early fungal colonization (Volpe et al., 2023). Our current observation of a comparable promotion of AM colonization under field conditions confirms CO bioactivity and efficiency also in an agricultural context, short of the presence of natural uncontrollable environmental variables, and represents a major advancement toward the use of CO as biostimulants in sustainable agriculture.

### Boosting AM colonization, productivity and plant community composition

In more detail, our results showed that the frequency of AM colonization in plant roots was significantly higher in the meadow treated with a combination of CO and a commercial microbial inoculum (MYC+CO) compared to both the untreated meadow (CTR) and the meadow treated only with the commercial inoculum (MYC), and this increase was most noticeable during the first months following the treatment (April 2017 sampling).

This booster effect of CO application on AM development was not mirrored on the increase of aboveground biomass. A significantly higher productivity was in fact recorded in April 2017 for both MYC+CO and MYC meadow compared to control conditions, the higher average value of MYC+CO samples not being significantly different from the MYC samples. The increase in plant biomass production upon AM inoculation, known as the ‘growth effect’, is related to both the direct improvement of plant nutrition by AM fungi and - particularly in natural and agronomical ecosystems - the establishment of synergistic interactions with other beneficial microorganisms, such as plant-growth promoting rhizobacteria (PGPRs), nitrogen fixing and phosphate-solubilizing bacteria (Raklami et al., 2019). Furthermore, a faster plant development and/or a greater vegetation density can be the consequence of the improved root system development (Kalamulla et al., 2022) and better seedling establishment in inoculated fields (van der Heijden, 2004; Zhang et al., 2012).

Plant community composition was also affected by our treatments: MYC and MYC+CO meadows showed significantly different species assemblage and a significantly higher species evenness compared to control in April 2017. A lower percentage cover of grasses (especially *Festulolium*), which instead dominated the control, was observed in both inoculated meadows, in favor of a higher cover of forb species, such as *Medicago sativa* and *T. pratense*. This observation is consistent with previous studies establishing that AM colonization tends to increase plant species diversity when the dominant species in the community is a weakly mycotrophic plant, such as annual species and C3 grasses (e.g., *Festulolium*), by increasing the competitive ability of the subordinate species that generally are represented by perennial forbs and legumes such as *M. sativa* and *T. pratense* (Karanika et al., 2008; Bahadur et al., 2019).

Moreover, the increased belowground competitiveness of legume species may generate a positive feedback in the aboveground competition, especially in the case of *M. sativa*, whose extensive canopy can reduce light access by neighboring species (Klabi et al., 2014). Weed suppression through increased functional dispersion represents an additional benefit to the multiple ecosystem services of forage mixtures for sustainable grassland production. Such benefits can arise from positive species interactions and complementary use of resources (Nyfeler et al., 2011; Hoekstra et al., 2016), suggesting that high-yielding mixtures capture and transform increased amounts of resources into biomass (van Ruijven and Berendse, 2005). Moreover, Suter et al. (2017) found that functionally diverse grass-legume mixtures can also have direct effects on plant community composition by reducing weed biomass and survival, and consequently the need for herbicide use, in particular when the mixture includes species of different rooting depth (as in the case of the shallow-rooted *Lolium perenne* and the deep-rooted *Trifolium pratense*). Furthermore, the fast development of the sown species in the first months after sowing is critical to reduce weed establishment and reproduction by limiting available resources (light, water, and minerals) (Weisberger et al., 2019), with a direct impact on the community composition over a longer period of time after meadow establishment.

The observed differences in the plant community structure had important implications for forage quality, so that, during the second year of the study, when the three meadows showed the greatest differences in terms of species composition, we found a significantly higher pastoral value in MYC+CO compared to MYC and control. This finding was consistent with the higher contribution of legumes and the lower contribution of weeds to the aboveground biomass of the MYC+CO meadow. Indeed, legume species generally show a higher Index of Specific Quality (ISQ) for their productivity, palatability, and preference by livestock (Cavallero et al., 2007). Furthermore, legumes enrich the forage protein content with positive effects on its quality (French, 2017). A similar effect was observed also for the grass species *Dactylis glomerata*, which in our study was found to be significantly associated with the MYC+CO meadow (French, 2017). Although we did not perform a chemical characterization of the forage, previous studies found increased P levels in forage from AM inoculated plants (Smith et al., 2003; Chippano et al., 2021).

### Applicative perspectives

AM fungal development is known to be subject to seasonal cycles that impact on spore germination and mycelial growth in the soil, as well as the colonization of host roots (Jakobsen et al., 2003; Kemmelmeier et al., 2022). Under this respect, the temporal extension of this study (i.e., 24 months) allowed us to investigate the effect of AM inoculation and CO treatment over a markedly longer period of time compared to previous studies. In this frame, while the early boost in AM colonization of MYC+CO plant roots was remarkable, a significant effect was also evident during the July-October 2017 period, while after our second CO application by spray, AM colonization levels were constantly and significantly higher in MYC+CO compared to both MYC and CTR samples but did not show any booster effect. This pattern is in line with the promotion of symbiosis development by exogenous CO application in controlled conditions (Volpe et al., 2020). Nevertheless, the observation of such a marked effect under field conditions - and over a period of several months after each treatment - is particularly significant and reinforces the conclusion that CO application has long term effects on AM symbiosis (Volpe et al., 2023).

The observed rise in AM colonization of MYC and CTR plants during the summer of the second year of this investigation (with root colonization of MYC plants reaching the same level as MYC+CO samples), warrants a distinct, yet equally significant, consideration. Most likely, this is the result of the revitalization of both native and inoculated AM propagules following the establishment of the mixed meadow after decades of monocultures (Johnson et al., 2005; Fester and Sawers, 2011). This effect of plant biodiversity on soil microbial population is well known but does not weaken the usefulness of either AM inoculation or CO application: the combination of these treatments at sowing can in fact support the early development and stabilization of symbiotic associations in the delicate period of meadow establishment.

Altogether, the combined impact of mixed sowing and mixed microbial inoculation on soil microbial activity - of which AM colonization was used here as a proxy - is very promising for regenerative and sustainable agricultural applications. In this scenario, the introduction of CO treatment has been a catalyst for AM development since the early months: a significant advantage for farmers, thanks to the positive effect of AM colonization on plant nutrition and health (Johnson et al., 2005).

Following this first study, focused on the plant community and a single group of beneficial microbes (AM fungi), it will now be very interesting to investigate the effect of microbial inoculation and CO treatment on the whole soil microbiota, through metagenomics analyses revealing the phylogenetic and functional composition of fungal and bacterial communities under each experimental condition. In this sense, it has already been demonstrated as the co-application of CO and AM fungi has an impact on the rhizosphere microecology, favoring the beneficial bacteria community and increasing soil microbial biomass carbon content (Ma et al., 2023). An intriguing aspect to be clarified is the effect of CO-dependent AM promotion on rhizobial infection in legumes, which host both symbionts in a largely unexplored physiological and metabolic balance.

Lastly, an analogous approach to the one we used can be developed for the restoration of natural ecosystems with grasslands, another promising field of application for AM symbiosis and plant growth-promoting microbes in general (Singh Rawat et al., 2022).

## Data availability statement

The raw data supporting the conclusions of this article will be made available by the authors, without undue reservation.

## Author contributions

LO: Conceptualization, Data curation, Formal Analysis, Investigation, Methodology, Software, Supervision, Writing – original draft, Writing – review & editing. VV: Conceptualization, Data curation, Formal Analysis, Investigation, Methodology, Supervision, Writing – original draft, Writing – review & editing. GC: Conceptualization, Data curation, Formal Analysis, Investigation, Methodology, Supervision, Writing – original draft, Writing – review & editing. MP: Data curation, Investigation, Writing – review & editing. EB: Data curation, Investigation, Writing – review & editing. AC: Data curation, Investigation, Writing – review & editing. CS: Conceptualization, Funding acquisition, Investigation, Methodology, Resources, Supervision, Visualization, Writing – original draft, Writing – review & editing. AG: Conceptualization, Funding acquisition, Investigation, Methodology, Project administration, Resources, Supervision, Validation, Visualization, Writing – original draft, Writing – review & editing.
