# Supplementary material for: Boosting species evenness, productivity and weed control in a mixed meadow by promoting arbuscular mycorrhizas
Source: Front Plant Sci. 2024 Feb 8;15:1303750. doi: 10.3389/fpls.2024.1303750 (PMC10883063; doi:10.3389/fpls.2024.1303750)
Supplement: Supplementary file 1 [file DataSheet_1.pdf]

Supplementary Material

Boosting species evenness, productivity and weed control in a mixed meadow by promoting arbuscular mycorrhizas.

Ludovica Oddi[1†], Veronica Volpe[1†], Gennaro Carotenuto[1†], Mara Politi[1], Elena Barni[1], Andrea Crosino[1], Consolata Siniscalco[1], Andrea Genre[1*]

[†]These authors contributed equally to this work and share first authorship

[1]Department of Life Sciences and Systems Biology, University of Turin, Viale P.A. Mattioli 25, 10125 Turin, Italy

***Correspondence:**
Corresponding Author
andrea.genre@unito.it

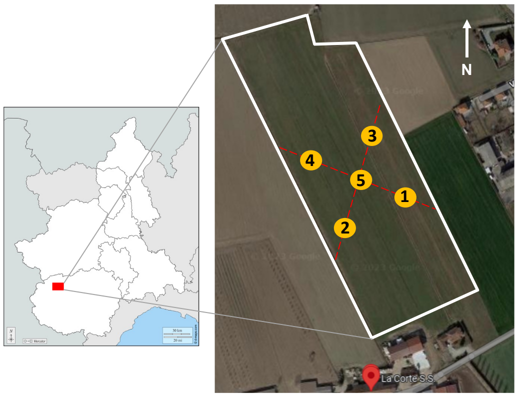

Sample	pH	Organic carbon (g/kg)	Organic matter (g/kg)	C/N	P_{ASS} (mg/kg)	N_{ASS} (%)	K_{scamb} (meq/100g)
1	6.49	20.85	35.95	8.0	72.32	0.26	0.21
2	6.40	18.37	31.67	7.1	59.00	0.26	0.16
3	6.23	19.25	33.18	7.1	51.66	0.27	0.16
4	6.13	21.60	37.24	7.2	71.25	0.30	0.19
5	6.30	22.07	38.05	7.4	69.80	0.30	0.23

Fig. S1 Experimental field location and soil analysis. The experimental field (kindly offered for this research by the owner farm 'La Corte') was located in Monasterolo di Savigliano, south-west Piedmont, Italy (Image by Google Maps; GPS coordinates: 44.6894147, 7.6196066). The yellow circles indicate the 5 points, distributed along the two diagonals (red dashed lines), where soil was sampled for chemical analysis and to evaluate the activity of native AM fungi. The chemical properties of soil, for each individual point, are shown in the table.

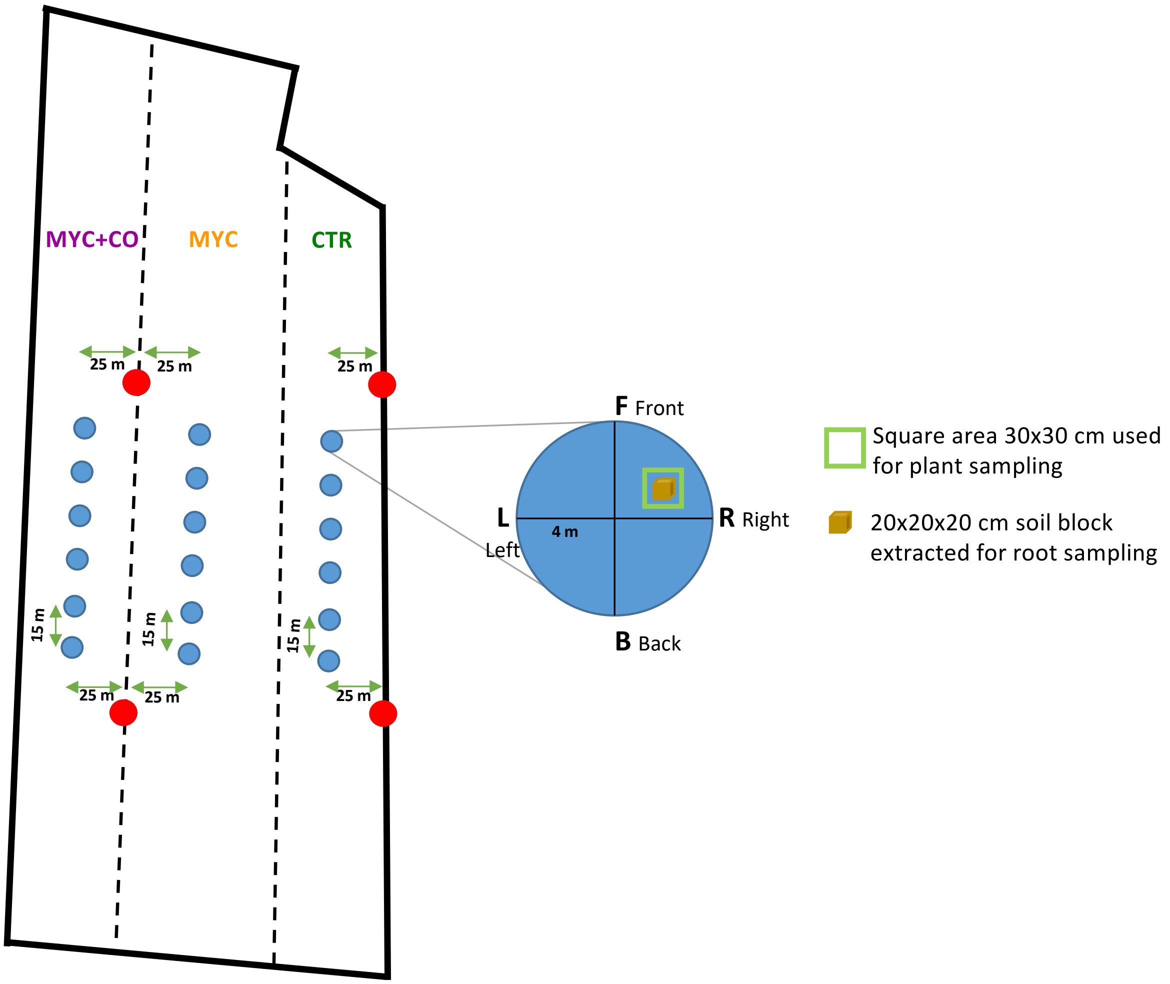

Fig. S2 Experimental set-up and sampling design. Sampling points (in blue) were distributed at regular distances (15 m) within the three experimental plots (CTR, MYC and MYC+CO) along a longitudinal transect, positioned at 25 m from fixed reference points (red). Circular (4m radius) sampling areas were centered on each sampling point and plant sampling was randomly distributed within the circular area by casting a 30x30cm wooden frame (green box, not in scale). Plant aerial parts were sampled within the wooden frame, using a battery-operated trimmer. Subsequently, roots were sampled by collecting a 20x20x20 cm soil block (brown) from underneath the trimmed area.

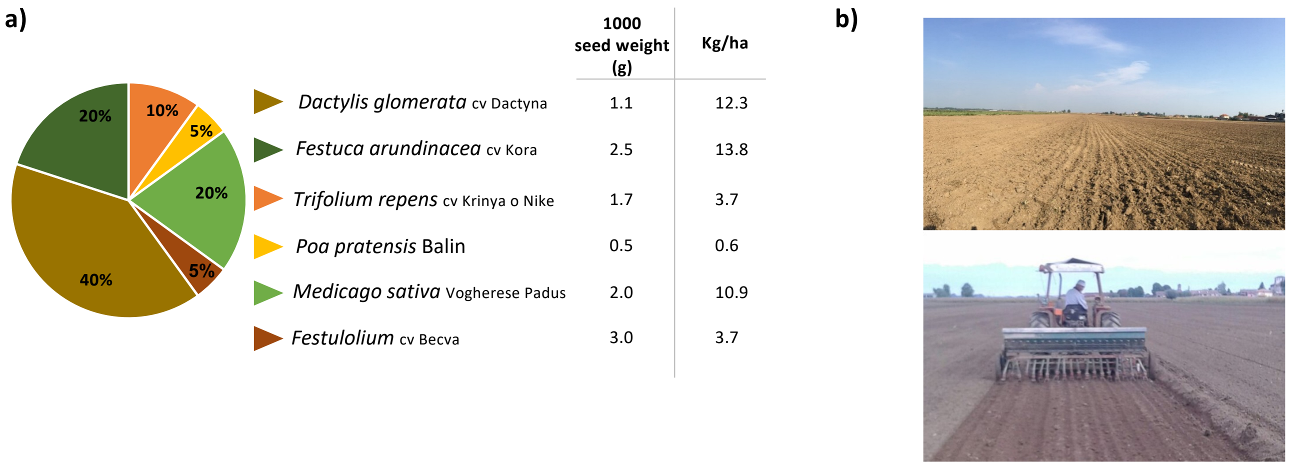

Fig. S3 Composition of the seed mixture and sowing. a) Percent composition in forage species within the seed mixture and amount (in kg/ha) used for sowing. b) Experimental field preparation and sowing in October 2016.

Quantification of AM colonization in *Medicago sativa*

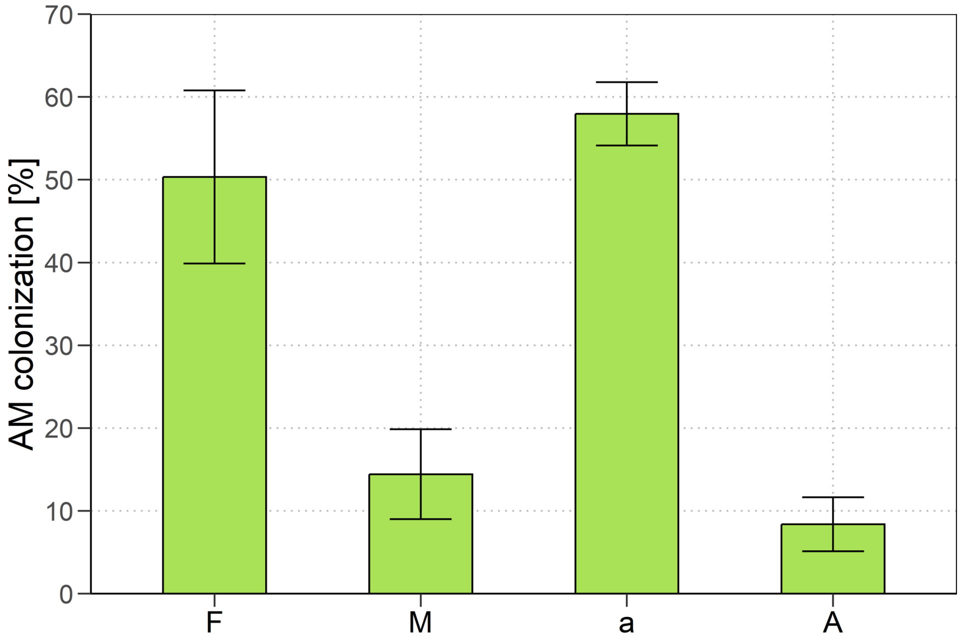

Fig.S4 Assesment of the native AM fungal activity in the experimental field soil. Microcosm analysis was performed by growing *Medicago sativa* in pots containing soil sampled from the experimental field. AM colonization was quantified according to Trouvelot et al. (1986), in terms of frequency of fungal structures in the root (F), intensity of colonization (M), arbuscule abundance within root fragments (a) and the whole root system (A). Bars represent ± SD.

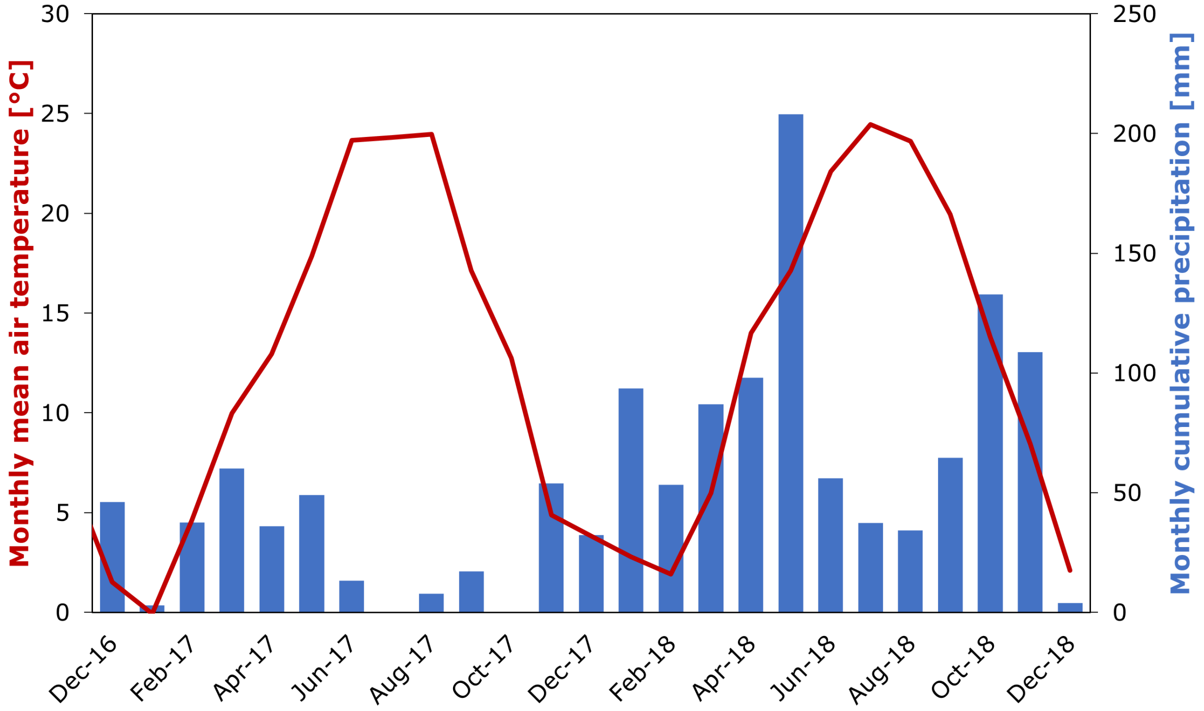

Fig. S5 Mean daily temperatures (in red; °C) and rainfall (in blue; mm) during the experimental period (December 2016 – October 2018).

Table S1. MYCOSAT F composition: AM fungi (40%), Rhizobacterial ($12.4x10^7$ CFU/g), the remaining percentage is represented by the other microorganisms.

Fungi			Bacteria	
**Arbuscular Mycorrhizal**	**Saprophytic**	**Ascomycetes**	**Rhizospheric**	**Actinomycetes**
Glomus spp. GB 67	*Trichoderma harzianum* TH 01	*Pichia pastoris* PP 59	*Bacillus amyloliquefaciens* BA 41	*Streptomyces spp.* SB 14
Glomus mosseae GP 11	*Pochonia chlamydosporia* PC 50		*Agrobacterium radiobacter* AR 39	
Glomus viscosum GC 41				

Table S2. Index of Specific Quality (ISQ) used to calculate the pastoral value (PV) of the meadows (Cavallero et al., 2007).

Plant species	Index of Specific Quality (ISQ)
Amaranthus sp.	*0*
Capsella bursa-pastoris	*0*
Dactylis glomerata	*4*
Digitaria sanguinaris	*0*
Echinocloa crus-galli	*0*
Festuca arundinacea	*3*
Festulolium	*4*
Lamium purpureum	*0*
Leontodon ispidus	*1*
Lolium italicum	*4*
Medigaco sativa	*3*
Onobrychis viciifolia	*2*
Poa pratensis	*4*
Poa trivialis	*2*
Portulaca oleracea	*0*
Rumex obtusifolius	*0*
Setaria viridis	*0*
Silene alba	*0*
Stellaria media	*0*
Taraxacum officinale	*2*
Trifolium pratense	*4*
Trifolium repens	*4*
Veronica persica	*0*
